# *Pleurotus eryngii* Ameliorates Lipopolysaccharide-Induced Lung Inflammation in Mice

**DOI:** 10.1155/2014/532389

**Published:** 2014-04-01

**Authors:** Junya Kawai, Tsugunobu Andoh, Kenji Ouchi, Satoshi Inatomi

**Affiliations:** ^1^Mushroom Research Laboratory, Hokuto Corporation, 800-8 Shimokomazawa, Nagano 381-0008, Japan; ^2^Department of Applied Pharmacology, Graduate School of Medicine and Pharmaceutical Sciences, University of Toyama, Toyama 930-0194, Japan

## Abstract

*Pleurotus eryngii* (*P. eryngii*) is consumed as a fresh cultivated mushroom worldwide and demonstrated to have multiple beneficial effects. We investigated the anti-inflammatory effect of *P. eryngii* in mice with acute lung injury (ALI). Intranasal instillation of lipopolysaccharide (LPS) (10 **μ**g/site/mouse) induced marked lung inflammation (increase in the number of inflammatory cells, protein leakage, and production of nitric oxide in bronchoalveolar lavage fluid) as well as histopathological damage in the lung, 6 h after treatment. Mice administered heat-treated *P. eryngii* (0.3–1 g/kg, p.o. (HTPE)) 1 h before LPS challenge showed decreased pulmonary inflammation and ameliorated histopathological damage. These results suggest that HTPE has anti-inflammatory effects against ALI. Thus, *P. eryngii* itself may also have anti-inflammatory effects and could be a beneficial food for the prevention of ALI induced by bacterial infection.

## 1. Introduction

*Pleurotus eryngii* is an edible mushroom native to Europe. Its natural habitat is the dead roots of the weed* Eryngium campestre*.* P. eryngii *is cultivated widely, and its production has been increasing in Asia, including Japan [1].* P. eryngii* is considered to be a health food because it is low in fat and calories but rich in amino acids, vitamins, and dietary fiber.* P. eryngii* is also bioactive, with hypolipidemic [2], antitumor [3], antioxidant [4–6], and antiallergic activities [1]. In particular,* in vitro* studies have demonstrated that the antiallergic activity of* P. eryngii* is caused by the downregulation of allergy-related signaling proteins (including inflammation-related proteins) by inhibition of the nuclear factor of activated T cells, nuclear factor-kappa B (NF-*κ*B), and high-affinity immunoglobulin E receptor (Fc*ε*RI) mediated signaling in antigen-stimulated mast cells. However, whether* P. eryngii* has clinical effectiveness remains to be determined.

Acute respiratory distress syndrome is a result of acute inflammation of the lung and noncardiogenic pulmonary edema that often leads to multiorgan system failure and death [7, 8]. Lipopolysaccharide (LPS) is present in the outer membrane of Gram-negative bacteria. LPS can cause acute inflammation of the lung because of neutrophil recruitment and pulmonary edema [9, 10]. Intranasal instillation of LPS in mice (as an animal model of acute lung injury (ALI)) has been shown to result in the release of proinflammatory cytokines, which cause aggregation of inflammatory cells and, consequently, injury to lung tissue [11, 12]. There have been reports with regard to these mechanisms and the features of this model. Such reports have shown that LPS activates alveolar macrophages directly and stimulates neutrophils to migrate into the lung and that the proinflammatory mediators released from these inflammatory cells recruit lymphocytes to the lung [7, 13]. The critical feature of LPS-induced ALI is the destruction of vascular integrity and the subsequent upregulated permeability results in protein leakage and pulmonary edema [9, 10, 14]. It has also been reported that nitric oxide (NO) has important roles in the pathogenesis of ALI because inhibitors of NO synthase inhibit LPS-induced damage [9, 15–17].

The aim of this study was to examine the anti-inflammatory effect of* P. eryngii in vivo *using an LPS-induced ALI model in mice. We used the whole* P. eryngii* to illustrate the functional utility of this mushroom as food.

## 2. Materials and Methods

### 2.1. Animals

Male BALB/c mice (6 weeks of age: Japan SLC, Ltd., Shizuoka, Japan) were used. They were kept under controlled temperature (21–23°C) and humidity (45–65%). The room was lit from 7:00 am to 7:00 pm and during the behavioral test. Food and water were available* ad libitum*. The study was approved by the Committee for Animal Experiments at the University of Toyama (Toyama, Japan).

### 2.2. Agents

LPS (*Escherichia coli 0111:B4*), dexamethasone, and pentobarbital sodium were purchased from Sigma-Aldrich (St. Louis, MO, USA). Other agents used in this study were purchased from Wako Pure Chemical Industries Ltd. (Osaka, Japan). LPS was dissolved in phosphate-buffered saline (PBS) and instilled intranasally. Dexamethasone, which was dissolved in saline with 10% ethanol, was given to mice in the dexamethasone group by intraperitoneal injection 1 h before LPS administration. Pentobarbital sodium was dissolved in saline containing 0.4% propylene glycol and 1.05% ethanol, and given by intraperitoneal injection.

### 2.3. Preparation of* P. eryngii* Intakes

**Figure 1:**
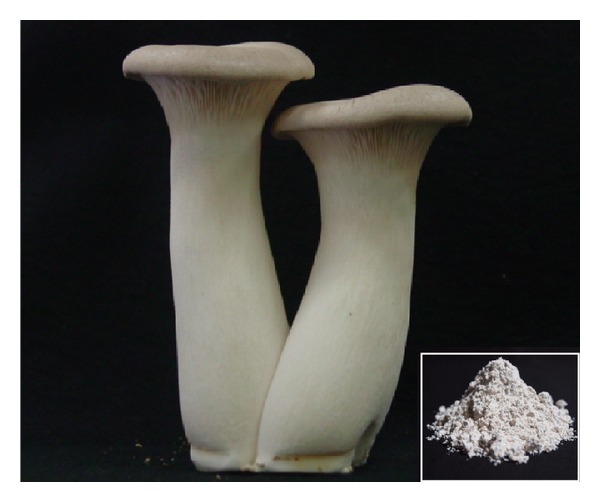
The fresh fruiting body of* Pleurotus eryngii* and freeze-dried powder of heat-treated* P. eryngii* (HTPE).

The fruiting body of* P. eryngii* (Figure 1) was obtained from Hokuto Co. (Nagano, Japan). It was then cut into small pieces and boiled in an equal amount of distilled water for 10 min. Heat-treated* P. eryngii* (HTPE) was freeze-dried and powdered. HTPE was resuspended in tap water and administered orally 1 h before intranasal administration of LPS.

### 2.4. LPS-Induced ALI Model in Mice

BALB/c mice were challenged with intranasal instillation (i.n.) of LPS (10 *μ*g in 50 *μ*L PBS per mouse) to induce lung inflammation. Control mice were given PBS (i.n.) without LPS. After 6 h, collection of bronchoalveolar lavage fluid (BALF) was carried out following the method of Chu et al. under anesthesia (sodium pentobarbital, 80 mg/kg, i.p.) [14]. After centrifugation (25 × g, 4°C, 5 min), cell pellets were resuspended in PBS for total cell counts using a hemacytometer. The supernatant was used for NO analyses and protein analyses.

### 2.5. Measurement for Protein Concentration

The protein concentration in BALF was determined using a protein assay kit (Bio-Rad Laboratories, Hercules, CA, USA).

### 2.6. Measurement for NO Production

A metabolite of NO, nitrite (NO^2−^), in BALF was measured using Griess reagent (1% sulfanilamide, 0.1% N-(1-naphthyl)ethylenediamine dihydrochloride, 2.5% phosphoric acid). Briefly, 50 *μ*L of BALF and 50 *μ*L of Griess reagent were mixed in a 96-well plate. The azo dye formed was determined with a spectrophotometer (Multiskan FC: Thermo Fisher Scientific K.K., Yokohama, Japan) at 540 nm using sodium nitrite as the standard.

### 2.7. Hematoxylin and Eosin (H&E) Staining

Anesthesia (sodium pentobarbital, 80 mg/kg, i.p.) was induced in mice 6 h after LPS treatment. Mice were then decapitated. Lungs were removed and, after washing with PBS, placed in 10% formalin solution. Preparation of paraffin-embedded sections and staining with H&E were undertaken using standard procedures. Staining was observed under a light microscope (AX80: Olympus, Osaka, Japan) with a charge-coupled device camera (Axio Cam; Carl Zeiss, Jena, Germany).

### 2.8. Immunohistochemical Staining

Anesthesia (sodium pentobarbital, 80 mg/kg, i.p.) was induced in mice 6 h after LPS treatment. Mice were then decapitated. Lungs were removed and, after washing with PBS, placed in 10% formalin solution. Preparation of paraffin-embedded sections and deparaffinization were undertaken using standard procedures. Deparaffinized sections were treated with methanol containing 0.3% hydrogen peroxide and then with 0.2% Triton X-100 in PBS. After treatment with 0.3% fetal bovine serum in PBS to block immunoglobulin binding, sections were incubated with rabbit anti-myeloperoxidase (MPO) antibody (DAKO, Glostrup, Denmark) or rat anti-Mac-2 antibody (Cedarlane, Ontario, Canada) at 4°C overnight, followed by horseradish peroxidase-conjugated anti-rabbit IgG antibody (DAKO) or horseradish peroxidase-conjugated anti-rat IgG antibody (DAKO). Color was developed using DAKO liquid with a 3,3-diaminobenzidine tetrahydrochloride (DAB) substrate chromogen system (DAKO) and counterstained with hematoxylin. These stained sections were observed under a light microscope (AX-80; Olympus) coupled to a CCD camera (Axio Cam; Carl Zeiss).

### 2.9. Statistical Analyses

Data are presented as the mean ± standard error of the mean (SEM). Statistical significance between groups was assessed using one-way analysis of variance and* post hoc* Holm-Sidak multiple comparisons. *P* < 0.05 was considered significant. Statistical analyses were done using Sigmaplot v11 (Systat Software, Inc., Chicago, IL, USA).

## 3. Results

### 3.1. Effects of HTPE on the Aggregation of Inflammatory Cells Induced by LPS in the Lung 

**Figure 2:**
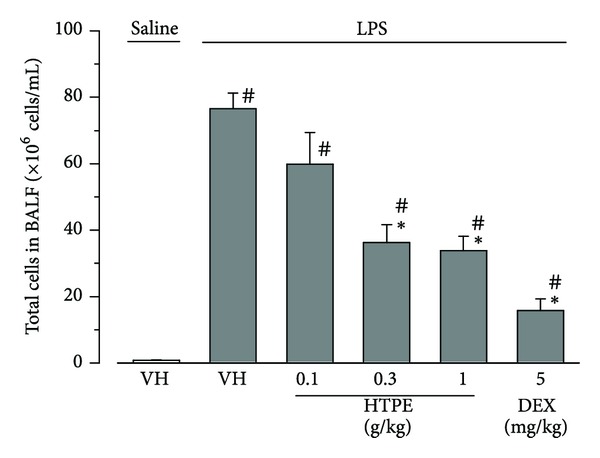
Effect of HTPE on the total number of cells in the BALF of LPS-induced ALI mice. Mice were administered, via the oral and intraperitoneal routes, HTPE (0.1–1 g/kg) and dexamethasone (DEX: 5 mg/kg, positive control group), respectively, 1 h before intranasal administration of LPS (10 *μ*g/site). Vehicle (VH: tap water, vehicle for HTPE) was also administered orally 1 h before intranasal administration of LPS (LPS group) or saline (vehicle for LPS, negative control group). BALF was collected 6 h after LPS challenge to measure the total number of cells. Values are presented as the mean and SEM (*n* = 6 for VH or HTPE; *n* = 4 for DEX). ^#^ *P* < 0.05 versus negative control group (VH + saline), *P < 0.05 versus LPS group (VH + LPS) (Holm-Sidak multiple comparisons).

LPS-induced ALI resulted in an increase in the number of inflammatory cells, such as neutrophils and macrophages, in the lung [14]. Six hours after intranasal instillation of LPS, the number of inflammatory cells in BALF increased significantly compared with intranasal instillation of saline (vehicle for LPS) (Figure 2). Oral pretreatment with HTPE (0.1–1.0 g/kg) significantly inhibited the LPS-induced increase in the number of cells in BALF compared with that of the vehicle (for HTPE) pretreated group (Figure 2). Intraperitoneal pretreatment with dexamethasone (5 mg/kg) also significantly decreased the number of cells in the BALF of LPS-treated mice compared with that of the vehicle (for HTPE) pretreated group (Figure 2).

### 3.2. Effect of HTPE on LPS-Induced Vascular Permeability in the Lung

**Figure 3:**
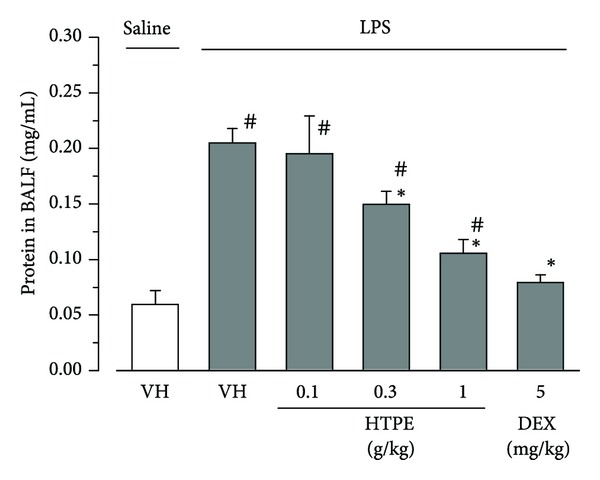
Effect of HTPE on total protein concentration in the BALF of LPS-induced ALI mice. Mice were administered, via the oral and intraperitoneal routes, HTPE (0.1–1 g/kg) and dexamethasone (DEX: 5 mg/kg, positive control group), respectively, 1 h before intranasal administration of LPS (10 *μ*g/site). Vehicle (VH: tap water, vehicle for HTPE) was also administered orally 1 h before intranasal administration of LPS (LPS group) or saline (vehicle for LPS, negative control group). BALF was collected 6 h after LPS challenge to measure total protein concentration. Values are presented as the mean and SEM (*n* = 6 for VH or HTPE; *n* = 4 for DEX). ^#^ *P* < 0.05 versus negative control group (VH + saline), *P < 0.05 versus LPS group (VH + LPS) (Holm-Sidak multiple comparisons).

One of the major pathological changes observed in LPS-induced ALI is increased vascular permeability, which results in increased protein leakage in BALF [14]. Six hours after intranasal instillation of LPS, the concentration of protein in BALF increased significantly compared with the intranasal instillation of saline (vehicle for LPS) (Figure 3). Oral pretreatment with HTPE (0.1–1.0 g/kg) significantly inhibited LPS-induced protein leakage in BALF compared with that of the vehicle (for HTPE) pretreated group (Figure 3). Intraperitoneal pretreatment with dexamethasone (5 mg/kg) also significantly attenuated the concentration of protein in the BALF of LPS-treated mice compared with that of the vehicle (for HTPE) pretreated group (Figure 3).

### 3.3. Effect of HTPE on LPS-Induced NO Production in the Lung. 

**Figure 4:**
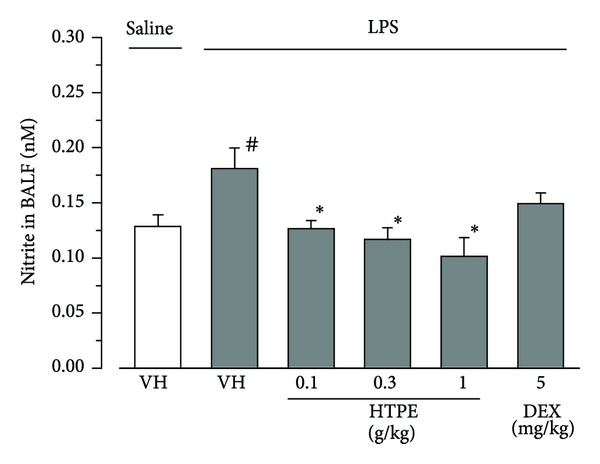
Effect of HTPE on production of nitric oxide in the BALF of LPS-induced ALI mice. Mice were administered, via the oral and intraperitoneal routes, HTPE (0.1–1 g/kg) and dexamethasone (DEX: 5 mg/kg, positive control group), respectively, 1 h before intranasal administration of LPS (10 *μ*g/site). Vehicle (VH: tap water, vehicle for HTPE) was also administered orally 1 h before intranasal administration of LPS (LPS group) or saline (vehicle for LPS, negative control group). BALF was collected 6 h after LPS challenge to measure the nitrite (a metabolite of nitric oxide) concentration. Values are presented as the mean and SEM (*n* = 6 for VH or HTPE; *n* = 4 for DEX). ^#^ *P* < 0.05 versus negative control group (VH + saline), *P < 0.05 versus LPS group (VH + LPS) (Holm-Sidak multiple comparisons).

It has been reported that NO plays an important part in the pathogenesis of ALI [8, 15–17]. Six hours after intranasal instillation of LPS, the concentration of nitrite and metabolites of NO in BALF increased significantly with intranasal instillation of saline (vehicle for LPS) (Figure 4). Oral pretreatment with HTPE (0.1–1.0 g/kg) significantly inhibited LPS-induced NO production in the lung compared with that of the vehicle (for HTPE) pretreated group (Figure 4). Intraperitoneal pretreatment with dexamethasone (5 mg/kg) showed a tendency toward inhibition of LPS-induced NO production, but not in a significant manner (*P* = 0.051) (Figure 4).

### 3.4. Effect of HTPE on LPS-Induced Pulmonary Histopathological Changes

**Figure 5:**
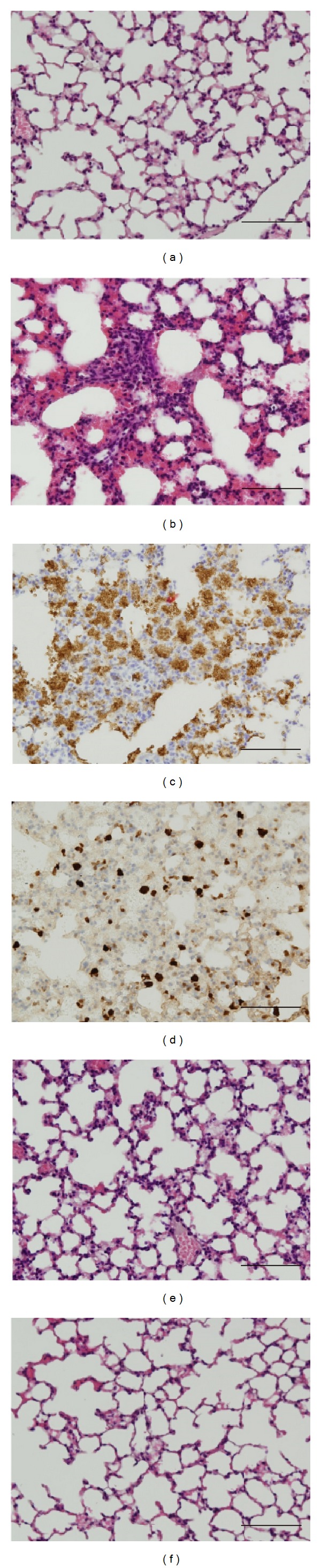
Effect of HTPE on histological changes in the lungs of LPS-induced ALI mice. Mice were administered, via the oral and intraperitoneal routes, HTPE (1 g/kg) and dexamethasone (DEX: 5 mg/kg, positive control group), respectively, 1 h before intranasal administration of LPS (10 *μ*g/site). Vehicle (tap water, vehicle for HTPE) was also administered orally 1 h before intranasal administration of LPS (LPS group) or saline (vehicle for LPS, saline group (negative control group)). Lungs (*n* = 3-4) from each experimental group were processed for histological evaluation 6 h after LPS challenge. The result in each group was almost identical. Lung sections were stained with hematoxylin and eosin ((a), (b), (e), and (f)). Lung sections in (c) and (d) show the immunoreactivity of myeloperoxidase (a marker of neutrophils) and Mac-2 (a maker of macrophages). Scale bar: 100 *μ*m. (a) Saline group (negative control group); ((b), (c), and (d))LPS group; (e) HTPE-treated LPS group; (f) DEX-treated LPS group (positive control group).

To evaluate the histopathological changes in LPS-treated mice, lung sections 6 h after LPS treatment were stained with H&E. Normal pulmonary histology was observed in mice that underwent intranasal instillation of saline (vehicle for LPS) (Figure 5(a)). LPS-treated lungs exhibited an apparent increase in infiltration of inflammatory cells, interstitial edema, and hyperemic thickening of the alveolar wall (Figure 5(b)). In particular, the infiltrated inflammatory cells in LPS-treated lung were neutrophils (MPO-immunoreactive cells) (Figure 5(c)) and macrophages (Mac-2-immunoreactive cells) (Figure 5(d)). However, these histopathological changes in lungs treated with LPS were ameliorated by pretreatment with HTPE (1 g/kg) (Figure 5(e)) or dexamethasone (5 mg/kg) (Figure 5(f)).

## 4. Discussion

We evaluated the anti-inflammatory activities of HTPE usinga LPS-induced model of ALI in mice. Pretreatment with HTPE as well as dexamethasone decreased the number of total cells, protein leakage, and NO production in the BALF of LPS-induced ALI mice. Histopathological studies revealed infiltration of inflammatory cells (such as neutrophils and macrophages), interstitial edema, and thickening of the alveolar walls in the lungs of LPS-induced ALI mice. These histopathological changes were also prevented in mice given HTPE and dexamethasone. These results suggest that HTPE improves the lung injury induced by LPS in mice through inhibition of the recruitment of inflammatory cells and overproduction of NO.

Mushrooms are low in calories, abundant in amino acids, vitamins, and dietary fiber, and are popular foods worldwide. It has been reported that some edible mushrooms have a wide range of pharmaceutical properties, including anti-inflammatory and antioxidant activities [4–6, 18–20].

It is well known that* P. eryngii* contains mainly *β*-glucans, including *β*-(1,3)-(1,6)-glucans [21, 22]. The* P. eryngii* (per 100 g of fresh fruiting body) used in the present study contained *β*-glucan (1.9 g). Polysaccharides such as *β*-glucans are known to be biologically active substances [23–25]. *β*-Glucans have been reported to possess immunomodulatory/immunostimulatory activities, and *β*-glucans usually have a *β*-(1,3)-linked main chain and *β*-(1,6)-linked branches [23, 24, 26, 27]. The frequency of branching varies, and immunomodulatory/immunostimulatory activities are dependent upon the structure of *β*-glucans [28, 29]. It has been reported that *β*-(1,6) branches could contribute to the stimulatory activity of *β*-glucans [28]. In the present study, we did not have information on the function and structure of *β*-glucans. However,* P. eryngii* contains *β*-(1,3)-(1,6)-glucans [21, 22]. Lentinan is a *β*-glucan from the fruiting bodies of* Lentinus edodes* and a *β*-(1,3)-glucan with *β*-(1,6) branching. Lentinan has been shown to suppress LPS-induced secretion of NO and tumor necrosis factor-*α* from RAW264.7 macrophages [25]. Thus, *β*-(1,3)-(1,6)-glucans in HTPE may have important roles in anti-inflammatory actions and NO production in lungs treated with LPS. In the present study, nitrite concentration was correlated with total cell numbers in BALF (*r* = 0.716, *P* ≤ 0.001). However, the decrease in nitrite concentration by HTPE was not completely correlated with the decrease in total cell numbers. *β*-Glucans inhibit LPS-induced NO production [25]. Thus, it is suggested that the decrease in nitrite concentration by HTPE is associated with the decrease in total cell numbers and inhibition of NO production by HTPE in inflammatory cells. The* P. eryngii* (per 100 g of fresh fruiting body) used in the present study also contained ergosterol (45.5 mg) and vitamin D2 (1.9 *μ*g). The fungal sterol ergosterol (known as provitamin D2) is abundant in mushrooms, as is its peroxide [30, 31]. Sterols suppress LPS-induced inflammatory responses in RAW264.7 macrophages through inhibition of the transcriptional activity of NF-*κ*B and CCAAT-enhancer-binding protein (C/EBP) *β* as well as phosphorylation of mitogen-activated protein kinases (MAPKs) [31]. Supplementation with vitamin D results in reductions in the levels of proinflammatory cytokines such as interleukin (IL)-4, IL-5, and IL-13 in the BALF of mice challenged with ovalbumin [32, 33]. Moreover, it has been reported that* P. eryngii* has a significantly higher amount of total phenolic compounds and has a close relationship with antioxidant activity and 2,2-diphenylpicrylhydrazyl-scavenging activity [6]. Taken together, these findings suggest that the components of* P. eryngii* may contribute to the anti-inflammatory actions of HTPE* in vivo*.

We used HTPE obtained from the whole fresh fruiting bodies of* P. eryngii*. HTPE (0.3–1.0 g/kg) was effective against ALI in mice. HTPE at 0.3–1.0 g/kg is equivalent to a fresh fruiting body at 3–10 g/kg. The effective dose in mice cannot be applied directly for humans, but a fresh fruiting body of* P. eryngii* (180–600 g per person (60 kg body weight)) should be eaten to protect against ALI. We consider that intake of* P. eryngii *(which has anti-inflammatory effects and which many people can obtain) on a daily basis could be a useful prevention strategy against ALI.

## 5. Conclusion

HTPE obtained from the heat-treated fresh fruiting body of* P. eryngii* prevented LPS-induced ALI through inhibition of infiltration of inflammatory cells, destruction of vascular integrity, and overproduction of NO and led to alleviation of histopathological damage. Our study supports the hypothesis that* P. eryngii* could be a beneficial food for the prevention of ALI associated with bacterial infection.
